# Toxicity of the insecticide sulfoxaflor alone and in combination with the fungicide fluxapyroxad in three bee species

**DOI:** 10.1038/s41598-021-86036-1

**Published:** 2021-03-25

**Authors:** C. Azpiazu, J. Bosch, L. Bortolotti, P. Medrzycki, D. Teper, R. Molowny-Horas, F. Sgolastra

**Affiliations:** 1 Dipartimento di Scienze e Tecnologie Agro-Alimentari, Alma Mater Studiorum Università di Bologna, Viale Fanin 42, 40127 Bologna, Italy; 2 CREAF, Universitat Autònoma de Barcelona, 08193 Bellaterra, Spain; 3 CREA-Consiglio per la Ricerca in Agricoltura e l’Analisi dell’Economia Agraria, Centro di Ricerca Agricoltura ed Ambiente, Via Corticella 133, 40128 Bologna, Italy; 4 Apiculture Division, Research Institute of Horticulture, 2A Kazmierska St., 24100 Puławy, Poland

**Keywords:** Conservation biology, Environmental sciences

## Abstract

The sulfoximine insecticide sulfoxaflor is regarded as a potential substitute for neonicotinoids that were recently banned in the EU due to their side effects on bees. Like neonicotinoids, sulfoxaflor acts as a competitive modulator of nicotinic acetylcholine receptors. In agricultural environments, bees are commonly exposed to combinations of pesticides, and neonicotinoids are known to interact synergistically with fungicides. The objective of our study is to assess the acute oral toxicity of sulfoxaflor alone and in combination with a single dose of fluxapyroxad, a succinate dehydrogenase inhibitor (SDHI) fungicide, in three bee species: *Apis mellifera*, *Bombus terrestris* and *Osmia bicornis*. Because synergism may be dose-dependent, we tested a range of sulfoxaflor doses. Synergistic effects were assessed using three different approaches: Bliss criterion of drugs independence, ratio test comparing LD_50_s and model deviation ratio. *Osmia bicornis* was the most sensitive species to sulfoxaflor and both *O. bicornis* and *A. mellifera* showed significant synergism between the insecticide and the fungicide. For the most part, these synergistic effects were weak and only occurred at early assessment times and intermediate sulfoxaflor doses. The potential ecological relevance of these effects should be confirmed in field and/or cage studies. Overall, our laboratory results demonstrate that sulfoxaflor is somewhat less toxic than the recently banned neonicotinoids imidacloprid, thiamethoxam and clothianidin, but much more toxic than other neonicotinoids (acetamiprid, thiacloprid) still in use in the EU at the time this study was conducted.

## Introduction

Anthropocene, the current geological epoch, is experiencing dramatic declines in insect abundance and diversity worldwide^1–3^. Among the different insect groups, bees (Hymenoptera: Apoidea: Anthophila), comprising about 20,000 species^4^, are particularly at risk^5–7^. Bees play a key role in ecosystem functioning ^8^ and provide an essential ecosystem service in the form of pollination^9^. The causes of bee decline are various, but agricultural intensification is recognized as one of the main drivers^2,10,11^. Agricultural intensification not only reduces the availability and quality of floral and nesting resources but also exposes bees to several toxic plant protection products^10^.

Before being authorized for commercial use, plant protection products undergo a stringent risk assessment process. Therefore, in theory, these products should be safe for bees, as long as they are applied following the producer's recommendations. However, current risk assessment procedures only test single compounds and do not account for the multiple-pesticide scenario to which bees are exposed in agricultural environments^12,13^. Exposure to multiple chemicals may lead to additive, antagonistic and synergistic interactions^14^. Importantly, the magnitude of these interactions is dose-dependent^14,15^. However, tests of synergism at field realistic dosages have only been conducted for a handful of pesticide combinations^16,17^. In addition, different bee species have different sensitivity to single pesticides^18–20^ and pesticide mixtures^21,22^ underscoring the need to include bee species other than *Apis mellifera* in pesticide risk assessment schemes^17^.

In this study, we assess the acute oral toxicity of the insecticide sulfoxaflor (SUL) at a range of concentrations, alone and in combination with a field-realistic dose of the fungicide fluxapyroxad (FLU), on two social (*A. mellifera*, *Bombus terrestris*) and one solitary (*Osmia bicornis*) bee species.

FLU is a pyrazole-carboxamide fungicide used on cereals and on many insect-pollinated crops, including citrus, pome fruits, and cucurbits^23^. FLU acts by inhibiting the succinate dehydrogenase (SDHI), a universal component of mitochondria that is highly conserved across living organisms^24^. With acute oral and contact LD_50_s of > 110.9 and > 100 µg/bee, respectively, FLU is considered non-toxic to honey bees^25^. In recent years this compound has experienced a rapid growth in the agricultural pesticide market^26^. SUL is a sulfoximine-based insecticide and, like neonicotinoids, it acts as an agonist of nicotinic acetylcholine receptors (nAChRs)^27,28^. This compound is relatively new and is regarded as a likely substitute for the neonicotinoid insecticides recently banned in the EU^17,29^. Some studies have shown that field-realistic doses of SUL affect egg-laying rates and reproductive success of bumblebees^30,31^, but not foraging and cognitive performance^32^. A semi-field study conducted with honey bees showed increased mortality during the exposure phase but no overall effects at the colony level^33^. Contact exposure to a mixture of SUL and the neonicotinoid insecticide imidacloprid resulted in synergistically increased mortality in honey bees^34^. By contrast, oral exposure to the same mixture caused significantly lower mortality than SUL alone^35^. This antagonistic interaction can be explained by the inhibitory effect of imidacloprid on feeding^35–38^. To our knowledge, no studies have assessed the toxicity of SUL in combination with a fungicide. Although fungicides are usually considered non-toxic to bees and thus are regularly applied during bloom, several studies have shown they can synergistically interact with neonicotinoid and pyrethroid insecticides^39^.

Our objective is to establish whether bees exposed to SUL are at risk and if this risk is exacerbated by the simultaneous exposure to a fungicide. We ask the following specific questions: (i) do the two compounds show synergistic effects? (ii) if so, are these effects dose-dependent? (iii) do the three bee species show different sensitivity to SUL and the SUL-FLU mixture? (iv) Are field-relevant SUL exposure levels toxic to these bee species?

## Results

Survival curves differed significantly among treatments in all three species (log-rank tests with survdiff function of the survival R package^40^: *A. mellifera*: df = 13, χ^2^ = 268.66, p < 2·10^–16^*; B. terrestris*: df = 13, χ^2^ = 357.78, p < 2·10^–16^; *O. bicornis*: df = 13, χ^2^ = 427.61, p < 2·10^–16^; see also Figs. S1-S3). Nevertheless, we found no significant differences between the solvent control and the FLU cumulative survival curves in any of the three bee species (Figs. S1-S3). On the other hand, a visual inspection of the survival curves (Figs. S1-S3) showed that the “toxic threshold” changed depending on the species (≥ 44 ng/bee in *A. mellifera*, ≥ 88 ng/bee in *B. terrestris*, ≥ 5.5 ng/bee in *O. bicornis*). This result is congruent with the different SUL LD_50_s obtained in the three bee species, showing that *O. bicornis* was the most sensitive species both when the toxicity endpoint is expressed in ng of SUL per bee and in ng per g of body weight (Table 1). *A. mellifera* showed higher sensitivity than *B. terrestris* when the LD_50_ is expressed in ng per bee but not when expressed in ng per g of body weight (Table 1).

**Table 1:** LD50s and 95% confidence limits (in parentheses) expressed in ng bee^−1^ and in ng g^−1^ of bee body weight following acute oral exposure to sulfoxaflor alone and in combination with fluxapyroxad at different assessment times (3, 24, 48, 72 and 96 h after exposure).

	LD_50_ *Apis mellifera* (ng/bee)		LD_50_ *Apis mellifera* (ng/g)		SR	
	Sulfoxaflor	Sulfoxaflor + fluxapyroxad	Sulfoxaflor	Sulfoxaflor + fluxapyroxad		
3 h	145.43 (109.07–202.21)	175.72 (130.87–251.45)	1788.80 (1341.60–2487.19)	2161.43 (1609.70–3092.91)	0.83	0.56–1.15
24 h	55.38 (26.34–111.46)	33.16 (15.18–64.28)	681.23 (324.03–1370.99)	407.82 (186.68–790.67)	1.67	0.79–5.68

	LD_50_ *Bombus terrestris* (ng/bee)		LD_50_ *Bombus terrestris* (ng/g)		S.R	
	Sulfoxaflor	Sulfoxaflor + fluxapyroxad	Sulfoxaflor	Sulfoxaflor + fluxapyroxad		
3 h	172.95 (131.10–216.09)	160.19 (125.95–199.02)	657.10 (498.10–821.00)	608.61 (478.55–756.16)	1.08	0.85–1.60
24 h	83.51 (68.69–98.21)	79.87 (63.88–94.87)	317.28 (260.97–373.15)	303.44 (242.72–360.45)	1.05	0.84–1.30
48 h	83.51 (68.69–98.21)	79.87 (63.88–94.87)	317.28 (260.97–373.15)	303.44 (242.72–360.45)	1.05	0.84–1.30
72 h	83.51 (68.69–98.21)	79.87 (63.88–94.87)	317.28 (260.97–373.15)	303.44 (242.72–360.45)	1.05	0.84–1.30
96 h	83.51 (68.69–98.21)	79.87 (63.88–94.87)	317.28 (260.97–373.15)	303.44 (242.72–360.45)	1.05	0.84–1.30

	LD_50_ *Osmia bicornis* (ng/bee)		LD_50_ *Osmia bicornis* (ng/g)		S.R	
	Sulfoxaflor	Sulfoxaflor + fluxapyroxad	Sulfoxaflor	Sulfoxaflor + fluxapyroxad		
3 h	81.21 (66.80–102.48)	59.65 (45.33–75.15)	885.65 (728.51–1117.52)	650.51 (494.28–819.49)	**1.36**	**1.03–2.21**
24 h	13.51 (10.41–16.69)	9.60 (7.75–11.54)	147.28 (113.55–182.00)	104.73 (84.46–125.87)	**1.41**	**1.07–2.03**
48 h	9.06 (6.80–11.19)	8.08 (5.92–10.08)	98.76 (74.20–122.04)	88.12 (64.59–109.94)	1.12	0.86–1.51
72 h	7.19 (4.12–9.73)	6.75 (3.60–9.43)	78.40 (44.90–106.15)	73.60 (39.23–102.82)	1.07	0.79–1.44
96 h	5.90 (1.97–9.28)	5.85 (2.01–9.10)	64.35 (21.49–101.22)	63.83 (21.97–99.19)	1.01	0.71–1.46

SR is the synergism ratio between the LD_50_ of sulfoxaflor alone and in combination with fluxapyroxad. Significant SRs (SR higher than 1 and the two confidence intervals do not overlap “1") are in bold. LD_50_ could not be determined for *Apis mellifera* at 48, 72 and 96 h because the Probit dose–response relationship at the tested doses was not significant.

**Figure 1:**
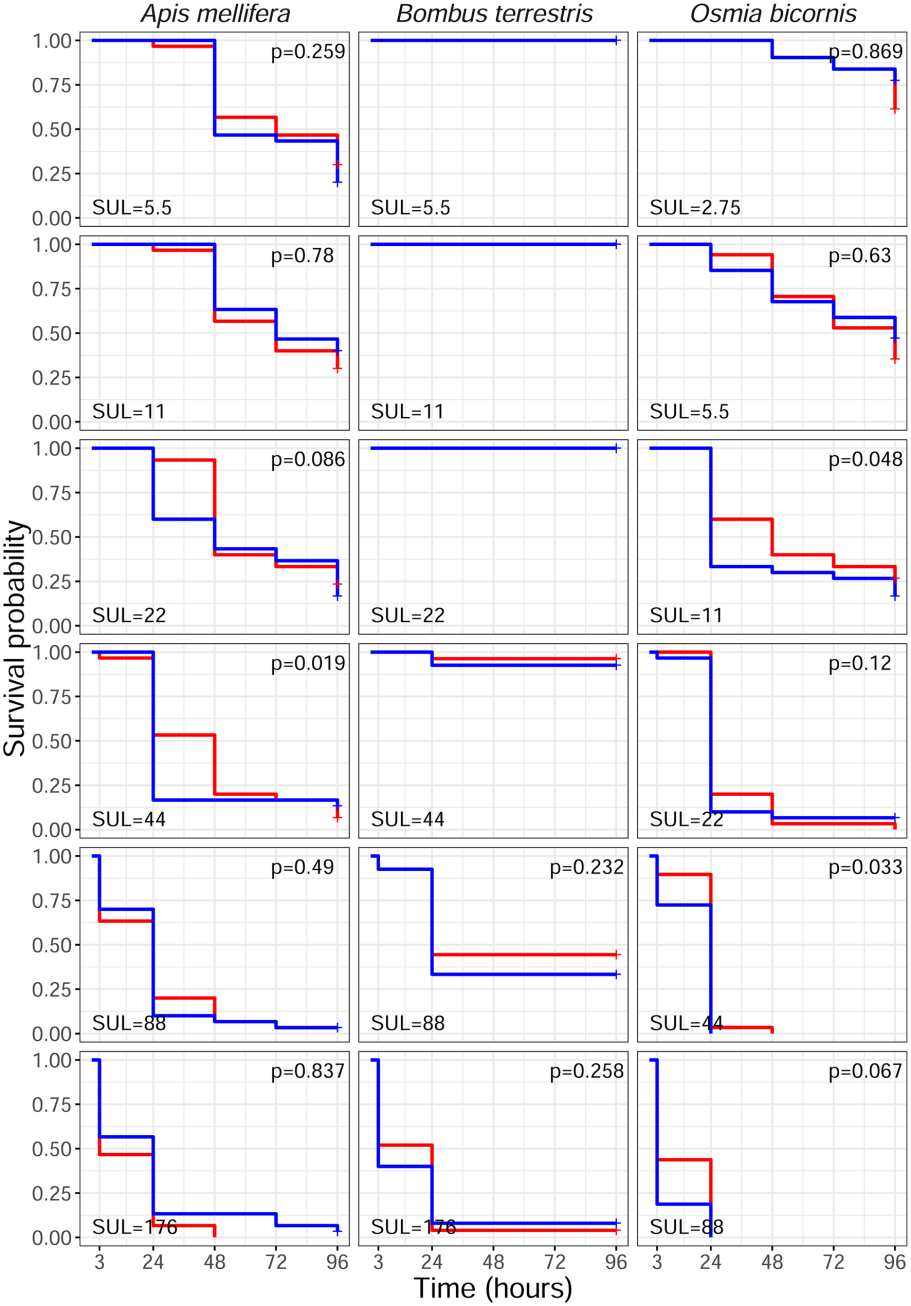
Observed (blue lines) and expected (red lines) effects of various doses (in ng bee^−1^) of sulfoxaflor (SUL) (y-axis) in combination with a single dose of fluxapyrozad (FLU) (1.2 µg/bee) on survival probability in three bee species. Significant synergism based on Bliss criterion of drugs independence (P < 0.05).

We followed three complementary approaches to assess synergism. Firstly, we compared the observed survival curve of the mixture treatment with the expected survival curve based on the Bliss criterion for drugs independence^41^. We found that the SUL + FLU combination synergistically decreased *O. bicornis* survival at 11 and 44 ng of SUL/bee and *A. mellifera* survival at 44 ng of SUL/bee (Fig. 1). No synergistic effects were observed in *B. terrestris* (Fig. 1). Secondly, we calculated the Synergism Ratio (SR) to compare the LD_50_ of SUL alone and in combination with FLU. LD_50_ values of SUL + FLU were significantly lower than those of SUL alone only in *O. bicornis* at 3 and 24 h after pesticide exposure (ratio test, Table 1). Third, we applied the toxic unit approach that showed synergism (Model Deviation Ratio MDR > 1.25) in *A. mellifera* (at 24 h) and in *O. bicornis* at 3 and 24 h (Table 2). This approach also showed slight antagonism in *A. mellifera* at 3 h after exposure (Table 2).

**Table 2:** Model Deviation Ratio (MDR) and Toxic Unit (TU) values at various assessment times in three bee species exposed to SUL alone and in combination with FLU.

Assessment times (h)	*Apis mellifera*			*Bombus terrestris*			*Osmia bicornis*		
	**MDR**	TU_SUL_	TU_SUL+FLU_	**MDR**	TU_SUL_	TU_SUL+FLU_	**MDR**	TU_SUL_	TU_SUL+FLU_
3	**0.82**	1.21	1.22	**1.07**	0.93	0.94	**1.34**	0.73	0.75
24	**1.64**	0.60	0.61	**1.03**	0.96	0.97	**1.39**	0.71	0.72
48	**–**	–	–	**1.03**	0.96	0.97	**1.11**	0.89	0.90
72	**–**	–	–	**1.03**	0.96	0.97	**1.05**	0.94	0.95
96	**–**	–	–	**1.03**	0.96	0.97	**1.00**	0.99	1.00

TU_FLU_ = 0.0108 (obtained by dividing the fluxapyrozad ingested dose (1.2 µg/bee) by its acute oral LD_50_ in *Apis mellifera* (110.9 µg/bee;^25^). TU_SUL_ is the ratio between the LD_50_ of sulfoxaflor alone and in combination. TU_SUL+FLU_ = TU_SUL_ + TU_FLU_; MDR = 1/TU_SUL+FLU_. MDR values between 0.83 and 1.25 indicate that combined toxicity follows the Dose/Concentration Addition model. MDR values < 0.83 or > 1.25 indicate antagonism and synergism between the two compounds, respectively^16^.

## Discussion

Neonicotinoids have been extensively used to protect crops and animals from insect pests since the 1990s. However, the development of pest resistance and their recent ban in EU prompted by the detection of side effects on bees, have raised the need to introduce new insecticides into the market as potential substitutes of these compounds^42,43^. Because SUL is one of the likely successors of neonicotinoids^29^, it is important to understand its potential effects on bees. The SUL LD_50_s obtained in our study for the three bee species (5.9–83.5 ng/bee) are somewhat higher than LD_50_s of neonicotinoids recently banned in the EU in 2018 (clothianidin: 1.17–3.12 ng/bee, thiamethoxam: 5 ng/bee and imidacloprid: 13–30 ng/bee^44,45^), but much lower than those of other neonicotinoids still in use in the EU at the time this study was conducted (acetamiprid: 14,530–22,200 ng/bee; thiacloprid: 17,320 ng/bee^46^). In addition, our results demonstrate that SUL may have negative effects even at lower doses if bees are simultaneously exposed to a fungicide (FLU).

Exposure to multiple pesticides is common in agricultural environments^12,13,47^ and synergism between insecticides and fungicides has been largely documented in bees (review in Carnesecchi et al.^16^). Several studies have reported synergism between EBI (ergosterol biosynthesis inhibitor) fungicides and insecticides (including neonicotinoids)^15,21,45,48–50^. However, studies showing synergism between SDHI fungicides and insecticides are less frequent likely because, based on their molecular mode of action, an interaction with the xenobiotic detoxification systems of insects is not expected^51,52^.

Our laboratory study reveals a weak but significant synergistic interaction between a SDHI fungicide (FLU) and an insecticide (SUL) with a similar mode of action to neonicotinoids. At the first two assessment times, co-exposure with FLU significantly reduced SUL LD_50_s in *O. bicornis* (synergism ratios: 1.36–1.41). A recent study calculated MDR values for 43 insecticide-fungicide combinations (Table S15 of Carnesecchi et al.^16^). Most insecticide-EBI fungicide combinations (23 of 30) showed synergism, but synergism was detected in only 2 of 7 insecticide-SDHI fungicide combinations. Our MDR values in *A. mellifera* (MDR_max_ = 1.64) and *O. bicornis* (MDR_max_ = 1.39) are close to the maximum MDR value obtained for insecticide-SDHI combinations (MDR = 1.45), but 10 times lower than the maximum values obtained for insecticide-EBI fungicide combinations (MDR = 10.00) in Carnesecchi et al.^16^. While the synergistic toxicity between insecticides and EBI fungicides in bees is likely due to the P450 enzyme inhibition^39^, the biochemical mechanisms underlying synergism between insecticides and SDHI fungicides are not well understood. Our study was conducted with a FLU-based commercial product, Sercadis. Therefore, a potential contribution of the co-formulants to the observed synergism cannot be excluded^17^.

In agreement with a previous study^15^, we found synergism to be dose-dependent. Thompson et al.^15^ showed that synergism increases with increasing fungicide dose. We tested different doses of insecticide with a single fungicide dose and found that synergism increased at intermediate insecticide doses in both *O. bicornis* (11 and 44 ng/bee) and *A. mellifera* (44 ng/bee). Lack of synergism at the lower and higher doses tested can be explained by the low toxicity of SUL at the lowest doses and the high toxicity of this compound (almost 100% mortality) at the highest doses . In addition, we found that the magnitude of synergism was dependent on the assessment time (synergism shortly after exposure; Tables 1 and 2). A previous study on *O. bicornis* yielded a similar result (Figs. 3 and 4 in^49^). Bees exposed to an insecticide-fungicide mixture experienced a strong decline in survival during the first hours after exposure. However, the longevity of those bees that survived the first hours of exposure to the mixture was similar to that of control bees, reflecting a high level of intra-population variability in sensitivity. In Sgolastra et al.^49^ this variability was partly explained by differences among individuals in emergence time (bees that took longer to emerge were more sensitive). However, all our bees emerged over two consecutive days and therefore other (unknown) factors should account for the observed intra-populational variability.

Comparison of pesticide sensitivity among the three bee species showed that *O. bicornis* was consistently more susceptible to SUL and SUL + FLU than the other two bee species, at all assessment times, both when expressed in ng of SUL per bee and in ng per g of bee body weight. This result is in agreement with previous studies and confirms that *Osmia* bees are more sensitive than honey bees and bumblebees to insecticides that target insect nAChRs^18,21,45,53^. Differences in P450 enzymes involved in xenobiotic detoxification could explain the different sensitivity to nAChR compounds between the bee families Apidae (*A. mellifera* and *B. terrestris*) and Megachilidae (*O. bicornis*)^54,55^. Probably owing to their large body size, bumblebees were less sensitive than honey bees, but when accounting for body weight, both species had similar sensitivity.

The LD_50_ value at 24 h of SUL obtained in our study for *A. mellifera* (55.4 ng/bee) was lower than the value reported in the dossier for the registration of this compound (146 ng/bee)^56,57^. This difference can be explained by differences in the age of the bees used (foragers and in-hive bees, respectively). It has been demonstrated that foragers are usually more sensitive to pesticides than in-hive bees^50^. The LD_50_ we obtained for *B. terrestris* (83.5 ng/bee) falls between the two values (27 and 150 ng/bee) reported in EFSA^55^ for two SUL formulated products. LD_50_ for *Bombus impatiens* is lower (19.4 ng/bee)^58^, probably due to the smaller size of this species.

The occurrence of synergistic effects between SUL and FLU also differed across bee species. We detected synergism in *A. mellifera* (at 24 h, MDR approach; and at 44 ng/bee, Bliss independence criterion approach) and *O. bicornis* (at 24 h, MDR and SR approaches; and at 11 and 44 ng/bee Bliss independence criterion approach). On the other hand, no synergism was observed in *B. terrestris* at any assessment time or SUL concentration. In the latter species, the MDR and SR (~ 1.00) indicate an additive effect between SUL and FLU.

Because SUL has only recently been introduced into the pesticide market, information on the levels of exposure under field conditions is scarce. However, studies conducted for the registration of this compound report SUL levels in the nectar of a variety of crops at various times after application (Appendix F of EPA document^56^). Based on this information, application of SUL commercial products, Closer and Transform, in the US is allowed up to 3 days before bloom for several crops, including pome fruits, stone fruits, canola and citrus. For other crops such as alfalfa and strawberry, label specifications advice users to notify local beekeepers or to spray when pollinators are least active (2 h prior to sunset or when temperatures are below 10 °C). Importantly, these label restrictions were established considering only honey bee endpoints and therefore do not necessarily protect other bees. Using the SUL levels reported in the EPA document^56^ and assuming a consumption of 80 mg of nectar with 15% sugar content^45,59^ we can compare the expected dose that a bee would consume during 1 h of foraging on crops treated with SUL with the LD_50_ of *O. bicornis*, the most sensitive of the three species tested in our study. In 5 of the 6 crops measured, including the two with no specific restrictions during bloom, the concentration of SUL in the nectar (≥ 0.073 mg/Kg; Table S1) would cause lethal effects in *O. bicornis* (Table S1). In peaches treated in pre-bloom, SUL residues would be toxic to *O. bicornis* even 5–7 days after application. These outcomes, based on theoretical calculations, should be confirmed in field or semi-field empirical studies. In addition to acute exposure and mortality, risk assessment should also consider chronic exposure and account for potential sublethal effects. A recent study shows that chronic exposure to SUL concentrations as low as 5 ng/g of sucrose solution has negative effects on reproduction of bumblebee colonies^30^.

Our laboratory study confirms that bee species differ in their sensitivity to pesticides and, more importantly, in their response to pesticide combinations. Based on the ratio between the lowest LD_50_ found for *A. mellifera* and *O. bicornis*, SUL is 10.6 times more toxic to *O. bicornis* per g of body weight. Although the interspecific differences observed in our study may partly be explained by differences in the protocols used for each species, these protocols were designed to mimic field conditions most likely to be encountered by each bee species in the field. In fact, differences among species in life history traits result not only in different sensitivity^45,54^, but also different exposure routes and levels^60–63^, thus hindering extrapolations across species^64^.

## Material and methods

### Bee populations and experimental procedures

For honey bees and bumblebees we followed, with some exceptions (see below), OECD standard toxicity protocols^65,66^. Standard protocols are not available for *Osmia* spp. Therefore, for *O. bicornis* we followed methods developed in previous studies^45,49^. The use of different protocols partly hinders the direct comparison across species. However, applying a unique protocol is impractical because the three bee species drastically differ in life history traits, in their response to laboratory conditions and in feeding behaviour^60^. For this reason, we decided to apply the most suitable methodology for each species.

We used three healthy, queen-right honey bee colonies (*A. mellifera ligustica*) located at the CREA-AA (Council for Agricultural Research and Economics–Agricultural and Environment Research Center), Bologna, Italy. These hives were managed following the guidelines for organic beekeeping (exclusively oxalic acid), with no treatment conducted in the 6 months preceding the study. In July 2019, we placed funnel traps in front of the hives to collect forager bees^67^. Following a previous study^45^, we chose to work with forager bees, instead of in-hive bees^65^ because they are more likely to be directly exposed to contaminated nectar and because a recent study showed that foragers are more sensitive to pesticides than in-hive bees^50^. Bees, anaesthetized for ∼30 min with a mixture of 60% CO_2_ and 40% synthetic air, were transferred in groups of 20 individuals to cardboard cages (9.5 × 6.5 × 5 cm). After a starvation period of ∼1 h, 200 μL of the test solution (sucrose 50% w/w) was provided to each group of bees using a common feeder (Eppendorf tube’s cap), assuming that, through trophallaxis, all individuals would ingest a similar dose (ca. 20 μL)^65^. The test solution was completely consumed within 1 h of exposure in all cages. Following the exposure phase, cages were maintained in an incubator in complete darkness at 25 ± 2 °C and 50–70% relative humidity for the duration of the test (96 h). We provided each cage with a 5 mL syringe filled with sucrose syrup for ad libitum feeding until the end of the experiment.

Six bumblebee colonies (*B. terrestris*) were purchased from BioPlanet s.c.a. (Cesena, Italy). Colonies contained 60–80 workers, brood in all stages of development and a laying queen. In October 2019, adult workers were collected under red light laboratory conditions and individually transferred to Nicot cages (7.1 × 2.0 cm). Very small (< 0.14 g) and very large (> 0.42 g) individuals were excluded^66^. Newly-emerged bees, recognisable by the greyish pubescence, and old bees, recognisable by sparse hairiness in the abdomen, were also excluded. Bees were acclimatised to the test conditions overnight (12–24 h) with ad libitum access to a sucrose solution. Throughout the test, they were maintained at 25 ± 2 °C and 50–70% relative humidity in continuous darkness. Prior to pesticide exposure, bees were starved for 3 h. Because *Bombus* do not perform trophallaxis and to avoid hierarchy fights among queen-less workers^66^, we used an individual feeding method whereby the test solution was offered through a 1 mL syringe inserted into the Nicot cage. Each individual was provided with 20 μL of test solution (sucrose 42% w/w) for an exposure period of 3 h. Feeders were visually inspected after the exposure phase, and only bees that consumed 100% of the test solution were used in the statistical analyses. Following the exposure phase, bees were maintained individually in the Nicot cages and fed ad libitum through a 2.5 mL syringe filled with sucrose syrup. To avoid confinement side effects, the Nicot cages of each treatment were placed side by side on a tray, so that workers could perceive their mutual presence.

*Osmia bicornis* individuals were obtained from a parent population reared in a pesticide-free area of the Kazimierz Landscape Park (Poland) and wintered from October 2018 at 3 °C at the Department of Agricultural and Food Sciences, University of Bologna, Italy. In May 2019, female cocoons were incubated at 23 °C until emergence and then transferred to a Plexiglas flight cage (50 × 50 × 50 cm) to allow them to deposit the meconium. We only used bees that emerged over two consecutive days during the peak of the emergence period (days 6–7)^38,49,68^. One day after emergence, these unmated, meconium-free females were individually housed in small plastic cylinders (width: 3.5 cm; height: 5.5 cm) with a transparent plastic lid. Since *Osmia* do not perform trophallaxis, we fed each bee individually with the ‘petal method’^45^, a modification of the ‘flower method’^69^. The test solution (20 μL) was pipetted into a tiny plastic ampoule (internal diameter 2 mm, external diameter 3 mm, height 5 mm) attached to a natural petal (*Euryops,* Asteraceae) inserted into a holder (diameter 1 cm, height 1 cm) made with salt paste. After ca. 3 h of exposure, bees that consumed 100% of the test solution were placed in groups of 3–5 individuals in larger plastic cages (width: 5.5–8 cm; height: 7 cm) with a transparent lid through which a 2.5 ml syringe filled with sucrose solution (sucrose 33% w/w) was inserted. These cages were kept in the laboratory at 23 ± 2 °C and 50–70% relative humidity under natural light.

### Treatments

For each bee species, we tested six doses of SUL in a geometric series (factor of 2), ranging from 2.75 to 176 ng/bee (concentration in the syrup solution: 0.14 to 8.8 mg/L) and a control (0 ng/bee). Each exposure to SUL was tested alone and in combination with a single dose of FLU (1.2 µg/bee).

SUL (purity 98%) was purchased from LGC (LGC Standards, Middlesex, UK) . The stock solution with a concentration of 0.88 mg SUL/mL acetone was used to prepare the test solutions. To obtain a range of appropriate concentrations based on the desired exposure level, the stock solution was first diluted in acetone until we reached final concentrations ranging from 0.014 to 0.88 mg/mL. These solutions were added to the feeding solution at a ratio of 10 μL/mL.

We used the commercial formulation of FLU (Sercadis, BAFS, Cesano Maderno, Italy). The tested concentration corresponds to the field application rate of Sercadis (300 g of a.i./L of commercial product) in orchards (20 mL/hL). To obtain this concentration, we prepared a stock solution with a FLU concentration of 3 g/L by dissolving Sercadis in distilled water. The stock solution was then diluted with the sugar solution at a ratio of 20 μL/mL to achieve the final concentration (60 mg/L).

Following the exposure phase, bees were fed ad libitum with sucrose solution. The final concentration of acetone in the feeding solution was adjusted to 1% (v/v) by adding pure acetone to all treatments not including SUL.

### Body weight

To minimise stress from manipulation, test bees were not weighed. Instead, 30 individuals of each species were randomly selected and weighed to obtain an average fresh body weight per species. Mean (± SD) body weights were 0.081 ± 0.015, 0.263 ± 0.049 and 0.092 ± 0.008 g in *A. mellifera*, *B. terrestris* and *O. bicornis*, respectively.

### Data analysis

Mortality was assessed 3 h after the end of the exposure phase and then checked every 24 h for 4 days. With these data we built Kaplan–Meier (K–M) survival curves for each species and compound concentration. Bees that were alive after 96 h were included as right-censored data. Then, a log-rank omnibus test was conducted to determine whether there were overall differences between treatments (survdiff function of the survival R package^40^ with $\rho =0$). Next, pairwise comparison of survival curves between treatments were carried out by means of pairwise tests (pairwise_survdiff function of the survminer R package^70^, with Holm multi-comparison corrections and $\rho =0$).

We followed three complementary approaches to test for synergism. The first approach was based on Bliss drugs independence criterion^41^. Bliss independence was tested for every single SUL + FLU combination with the FHtestrcc function of the FHtest R package^71^. We determined the expected survival curve, computed as:

1 $${S}_{{\text{Expected}}_{\text{SUL+FLU}}}\left(t\right)={S}_{{\text{Observed}}_{\text{FLU}}}\left(t\right)\cdot {S}_{{\text{Observed}}_{\text{SUL}}}\left(t\right)$$

where $S\left(t\right)$ is the probability for an individual to have survived longer than *t* .

Thus, if Bliss independence held, we would expect no differences between the expected and the observed survival curves (i.e. our null hypothesis ${H}_{0};$^41^):

2 $${{H}_{0}\equiv S}_{{\text{Expected}}_{\text{SUL+FLU}}}\left(t\right)={S}_{{\text{Observed}}_{\text{SUL+FLU}}}\left(t\right)$$

However, assuming that there could be a synergistic effect between the two components such that it would decrease survival of bees exposed to SUL + FLU, our alternative hypothesis ${H}_{1}$, was that the observed survival would be lower than the expected one:

3 $${{H}_{1}\equiv S}_{{\text{Expected}}_{\text{SUL+FLU}}}\left(t\right)>{S}_{{\text{Observed}}_{\text{SUL+FLU}}}\left(t\right)$$

Note that the definition of Bliss independence in Demidenko & Miller^41^ paper differs from our Eq. (2) because their study analysed increased survival following drug administration, whereas we analysed decreased survival following exposure to toxicants (E. Demidenko, pers. comm.).

Tests for each species and SUL concentration were then calculated to determine whether ${H}_{0}$ could be rejected. Since a visual inspection of survival curves indicated that differences between observed and expected curves were higher at early and/or middle time points, we selected a value of $\rho =1$, equivalent to the Peto and Peto log-rank test (see manual of the aforementioned FHtest R package for detailed information).

The second approach relied on a ratio test based on LD_50_ values. For each bee species, the LD_50_ values and their 95% confidence limits of SUL and SUL + FLU for each assessment time (3, 24, 48, 72 and 96 h) were determined after probit regression analysis using IBM SPSS Statistics 22.0.0.0 software (package for Windows, 64-bit edition, Chicago, USA). Then, a ratio test comparing the ratio of the LD_50_ of SUL and SUL + FLU respectively was performed. This test produces a synergism or antagonism ratio with the associated 95% confidence interval. Synergism occurs when the ratio is higher than 1 and the two confidence intervals do not overlap “1”^72^.

Finally, for our third approach we calculated the model deviation ratio (MDR) to determine if the SUL + FLU interaction caused synergistic (MDR > 1.25), additive (0.83 < MDR < 1.25), or antagonistic (MDR < 0.83) effects (refined thresholds for MDR in ^16^). MDR measures the deviations from the assumption of additivity following the concentration addition (CA) model^73^. MDR values are the ratio between the expected toxic unit (TU) (by definition = 1) and the observed TU for a binary mixture. To estimate MDR, we calculated the TU of each individual pesticide (SUL, FLU) and of the binary mixture (SUL + FLU). TU_FLU_ = 0.0108 (obtained by dividing the FLU ingested dose (1.2 µg/bee) by its acute oral LD_50_ in *Apis mellifera* (110.9 µg/bee;^25^). TU_SUL_ is the ratio between the LD_50_ of SUL alone and in combination in each assessment time. TU_SUL+FLU_ = TU_SUL_ + TU_FLU_; MDR = 1/TU_SUL+FLU_.

## Supplementary Information

Supplementary Information 1.

Supplementary Information 2.

Supplementary Information 3.

## Data Availability

Data available in the Supplementary information.
