# Supplementary material for: Toxicity of the insecticide sulfoxaflor alone and in combination with the fungicide fluxapyroxad in three bee species
Source: Sci Rep. 2021 Mar 25;11:6821. doi: 10.1038/s41598-021-86036-1 (PMC7994444; doi:10.1038/s41598-021-86036-1)
Supplement: Supplementary file 2 — Supplementary Information 2. [file 41598_2021_86036_MOESM2_ESM.docx]

**SUPPLEMENTARY INFORMATION FOR:**

**Toxicity of the insecticide sulfoxaflor alone and in combination with the fungicide fluxapyroxad in three bee species**

Azpiazu C.^1^, Bosch J. ^2^, Bortolotti L. ^3^, Medrzycki P. ^3^, Teper D. ^4^, Molowny-Horas R. ^2^, Sgolastra F. ^1^

^1^Dipartimento di Scienze e Tecnologie Agro-Alimentari, Alma Mater Studiorum Università di Bologna, viale Fanin 42, 40127, Bologna, Italy. ^2^CREAF, Universitat Autònoma de Barcelona, Cerdanyola del Vallès, 08193, Spain. ^3^CREA-Consiglio per la Ricerca in Agricoltura e l’Analisi dell’Economia Agraria, Centro di Ricerca Agricoltura ed Ambiente, Via di Saliceto 80, 40128, Bologna, Italy. ^4^Research Institute of Horticulture, Apiculture Division, 2 Kazmierska st., 24100, Puławy, Poland. Correspondence and requests for materials should be addressed to F.S. (email: fabio.sgolastra2@unibo.it)

|  |  |
| --- | --- |
|  |  |
|  |  |

**Figure S1**: Survival probability at different doses (in ng/bee) of sulfoxaflor (SUL) with and without fluxapyroxad (FLU) in *Apis mellifera*. Different letters denote significant differences (All Pairwise Multiple Comparison Procedures: Log-rank test). SC: solvent control. N.B.: the lines for SC and FLU are the same in each panel. Lines with the same letter are not significantly different.

|  |  |
| --- | --- |
|  |  |

**Figure S2:** Survival probability at different doses (in ng/bee) of sulfoxaflor (SUL) with and without fluxapyroxad (FLU) in *Bombus terrestris*. Different letters denote significant differences (All Pairwise Multiple Comparison Procedures: Log-rank test)). SC: solvent control. Survival curves at doses below 44 ng of sulfoxaflor are not shown because all bees were alive at 96 h. N.B.: the lines for SC and FLU are the same in each panel. Lines with the same letter are not significantly different.

|  |  |
| --- | --- |
|  |  |
|  |  |

**Figure S3**: Survival probability at different doses (in ng/bee) of sulfoxaflor (SUL) with and without fluxapyroxad (FLU) in *Osmia bicornis*. Different letters denote significant differences (All Pairwise Multiple Comparison Procedures: Log-rank test). SC: solvent control. N.B.: the lines for SC and FLU are the same in each panel. Lines with the same letter are not significantly different.

**Table S1:** Mean concentration of sulfoxaflor (SUL) in the hand-collected nectar of six crops measured on different days following foliar sprays. Data from figures provided in EPA (2019) extracted using [WebPlotDigitizer](https://automeris.io/WebPlotDigitizer) (<https://automeris.io/WebPlotDigitizer/>). Concentrations in red (≥0.07 mg/Kg) equal or exceed *O. bicornis* LD_50_ (5.90 ng/bee) when converted to ng of SUL ingested by a bee during one hour of foraging assuming a consumption of 80 mg of nectar with 15% sugar content (EFSA, 2013; Sgolastra et al., 2017).

| **Alfalfa.** Two applications (pre-bloom and bloom, respectively) at a rate of 0.09 lb a.i./acre. | | |  | **Canola**. Two applications (pre-bloom and bloom, respectively) at a rate of 0.023 lb a.i./acre. | | |  | **Peach.** One application at a rate of 0.09 lb a.i./acre in pre-bloom (plot 1 and 2), early bloom (plot 3 and 4) and full bloom (plot 5). | | |
| --- | --- | --- | --- | --- | --- | --- | --- | --- | --- | --- |
| Site | days | concentration (mg/kg) |  | Site | days | concentration (mg/kg) |  | Site/plot | days | concentration (mg/kg) |
| Hertford, NC | 0 | 34.14 |  | Northwood, ND | 1 | 0.10 |  | Hart, MI/3 | 0 | 0.19 |
| Live Oak, CA | 0 | 14.91 |  | Hood River, OR | 1 | 0.09 |  | Hart, MI/4 | 0 | 0.08 |
| Hertford, NC | 1 | 24.39 |  | Northwood, ND | 2 | 0.04 |  | Hart, MI/3 | 1 | 0.10 |
| Live Oak, CA | 1 | 2.33 |  | Hood River, OR | 2 | 0.06 |  | Hart, MI/4 | 1 | 0.06 |
| Hertford, NC | 2 | 6.79 |  | Northwood, ND | 7 | 0.00 |  | Hart, MI/5 | 1 | 0.03 |
| Live Oak, CA | 2 | 0.77 |  | Hood River, OR | 7 | 0.01 |  | Hart, MI/3 | 2 | 0.06 |
| Hertford, NC | 7 | 0.26 |  | Northwood, ND | 14 | 0.00 |  | Hart, MI/2 | 3 | 0.04 |
| Live Oak, CA | 7 | 0.02 |  | Hood River, OR | 14 | 0.00 |  | Hart, MI/4 | 3 | 0.02 |
| Hertford, NC | 14 | 0.00 |  |  |  |  |  | Hart, MI/2 | 4 | 0.13 |
| Live Oak, CA | 14 | 0.19 |  |  |  |  |  | Hart, MI/3 | 4 | 0.05 |
|  |  |  |  |  |  |  |  | Hart, MI/2 | 5 | 0.11 |
|  |  |  |  |  |  |  |  | Hart, MI/1 | 6 | 0.08 |
|  |  |  |  |  |  |  |  | Hart, MI/1 | 7 | 0.08 |
|  |  |  |  |  |  |  |  | Hart, MI/2 | 7 | 0.07 |
|  |  |  |  |  |  |  |  | Hart, MI/1 | 8 | 0.06 |
|  |  |  |  |  |  |  |  | Hart, MI/1 | 10 | 0.05 |
|  |  |  |  |  |  |  |  |  |  |  |
| **Pumpkin.** Two applications (pre-bloom and bloom, respectively) at a rate of 0.07 lb a.i./acre. | | |  | **Strawberry.** Two applications (pre-bloom and bloom, respectively) at a rate of 0.07 lb a.i./acre. | | |  | **Sunflower.** Two applications (pre-bloom and bloom, respectively) at a rate of 0.09 lb a.i./acre. | | |
| Site | days | concentration (mg/kg) |  | Site | days | concentration (mg/kg) |  | Site | days | concentration (mg/kg) |
| Belvidere, NC | 0 | 0.07 |  | Dover, FL | 0 | 0.84 |  | Stillwell, KS | 1 | 0.22 |
| Zamora, CA | 0 | 0.00 |  | Yuba City, CA | 0 | 8.98 |  | Stillwell, KS | 2 | 0.11 |
| Belvidere, NC | 1 | 0.04 |  | Dover, FL | 1 | 2.24 |  | Stillwell, KS | 4 | 0.03 |
| Zamora, CA | 1 | 0.00 |  | Yuba City, CA | 1 | 0.79 |  | Stillwell, KS | 4 | 0.02 |
| Belvidere, NC | 2 | 0.02 |  | Dover, FL | 2 | 0.46 |  | Stillwell, KS | 7 | 0.00 |
| Zamora, CA | 2 | 0.00 |  | Yuba City, CA | 2 | 0.54 |  |  |  |  |
| Belvidere, NC | 7 | 0.00 |  | Dover, FL | 7 | 0.04 |  |  |  |  |
| Zamora, CA | 7 | 0.00 |  | Yuba City, CA | 7 | 0.14 |  |  |  |  |
| Zamora, CA | 21 | 0.00 |  | Dover, FL | 14 | 0.05 |  |  |  |  |
|  |  |  |  | Yuba City, CA | 14 | 0.10 |  |  |  |  |

**References**

EFSA, 2013. European Food Safety Authority. Guidance on the risk assessment of plant protection products on bees (Apis mellifera , Bombus spp. and solitary bees). EFSA J. 11, 3295. doi:10.2903/j.efsa.2013.3295

EPA, 2019. Ecological Risk Assessment for the Registration Review of Sulfoxaflor. United States Environ. Prot. Agency.

Sgolastra, F., Medrzycki, P., Bortolotti, L., Renzi, M.T., Tosi, S., Bogo, G., Teper, D., Porrini, C., Molowny-Horas, R., Bosch, J., 2017. Synergistic mortality between a neonicotinoid insecticide and an ergosterol-biosynthesis-inhibiting fungicide in three bee species. Pest Manag. Sci. 73, 1236–1243. doi:10.1002/ps.4449
